# Speeding Up Non-Parametric Bootstrap Computations for Statistics Based on Sample Moments in Small/Moderate Sample Size Applications

**DOI:** 10.1371/journal.pone.0131333

**Published:** 2015-06-30

**Authors:** Elias Chaibub Neto

**Affiliations:** Sage Bionetworks, Seattle, Washington, United States of America; Technical University Darmstadt, GERMANY

## Abstract

In this paper we propose a vectorized implementation of the non-parametric bootstrap for statistics based on sample moments. Basically, we adopt the multinomial sampling formulation of the non-parametric bootstrap, and compute bootstrap replications of sample moment statistics by simply weighting the observed data according to multinomial counts instead of evaluating the statistic on a resampled version of the observed data. Using this formulation we can generate a matrix of bootstrap weights and compute the entire vector of bootstrap replications with a few matrix multiplications. Vectorization is particularly important for matrix-oriented programming languages such as R, where matrix/vector calculations tend to be faster than scalar operations implemented in a loop. We illustrate the application of the vectorized implementation in real and simulated data sets, when bootstrapping Pearson’s sample correlation coefficient, and compared its performance against two state-of-the-art R implementations of the non-parametric bootstrap, as well as a straightforward one based on a for loop. Our investigations spanned varying sample sizes and number of bootstrap replications. The vectorized bootstrap compared favorably against the state-of-the-art implementations in all cases tested, and was remarkably/considerably faster for small/moderate sample sizes. The same results were observed in the comparison with the straightforward implementation, except for large sample sizes, where the vectorized bootstrap was slightly slower than the straightforward implementation due to increased time expenditures in the generation of weight matrices via multinomial sampling.

## Introduction

Since its introduction, the bootstrap [1] has become, perhaps, the most popular statistical tool for assessing uncertainty of unknown quantities in situations were analytical solutions are not available, or modeling assumptions and asymptotic approximations are invalid. From a practical standpoint, the bootstrap is specially appealing because of its simplicity. In its most elementary format, the bootstrap corresponds to a distribution-free technique, referred as the non-parametric bootstrap, where a bootstrap replication is generated by simply evaluating the statistic of interest in a re-sampled version of the original data. Nonetheless, in addition to this basic non-parametric approach, a multitude of alternative bootstrap schemes have been proposed in the literature for accounting for model specific characteristics, including parametric and semi-parametric bootstrapping techniques [2, 3]. For instance, in regression models, several distinct approaches based on re-sampling of residuals have been proposed [2–4], in addition to the simple non-parametric bootstrap, denoted by paired or case bootstrap in this particular context.

In this paper we focus on the basic non-parametric bootstrap, and propose a vectorized implementation for statistics based on sample moments. Our approach is based on the multinomial sampling formulation of the non-parametric bootstrap. This formulation is described in the next section, but, in essence, follows from the fact that the bootstrap distribution of any sample moment statistic (generated by sampling *N* data points with replacement from a population of *N* values, and evaluating the statistic on the re-sampled version of the observed data) can also be generated by weighting the observed data according to multinomial category counts sampled from a multinomial distribution defined over *N* categories (i.e., data points) and assigning probability 1/*N* to each one of them.

The practical advantage of this multinomial sampling formulation is that, once we generate a matrix of bootstrap weights, we can compute the entire vector of bootstrap replications using a few matrix operations. The usual re-sampling formulation, on the other hand, is not amenable to such vectorization of computations, since for each bootstrap replication one needs to generate a re-sampled version of the data. Vectorization is particularly important for matrix-oriented programming languages such as R [5] and Matlab [6], where matrix/vector computations tend to be faster than scalar operations implemented in a loop.

Using Pearson’s correlation coefficient, we illustrate the gain in computational speed achieved by the vectorized bootstrap implementation in both real and simulated data sets. We compare our R implementation to state-of-the-art implementations provided in the bootstrap [7] and boot [3, 8] R packages, across different sample sizes and number of bootstrap replications. Because, the functions implementing the non-parametric bootstrap (in these packages) also handle additional functionality (e.g., jackknife after bootstrap, parametric bootstrap, etc), and go through a series data checks before starting the bootstrap computations, we also benchmark the vectorized implementation against a straightforward implementation based on a for loop. Our results show that the vectorized bootstrap compares favorably against the bootstrap and boot implementations in all cases tested, but is slightly slower than the for loop when the sample size is large (although it is much faster for small/moderate sample sizes). In order to clarify why this is the case, we also compared the time spent on the generation of the matrix of multinomial counts (from a call to the rmultinom function in the R base library) to the time spent in the generation of the bootstrap replications vector via matrix multiplications. Our study shows that sampling from the multinomial distribution is the time bottleneck when the sample size, *N*, is large.

This paper is organized as follows. The Methods section: (i) presents notation and background on the standard data re-sampling approach for the non-parametric bootstrap; (ii) describes the multinomial sampling formulation of the bootstrap; and (iii) explains how to vectorize the bootstrap calculations. The Results section reports a comparison of computation times required by the vectorized and standard approaches, when bootstrapping Pearson’s sample correlation coefficient in real and simulated data sets, as well as, the benchmarking of the multinomial counts sampling versus the matrix operations. Finally, the Discussion section presents some final remarks, points out that the Bayesian bootstrap computations can also be easily vectorized, and discusses the settings where the vectorized bootstrap is expected to produce the strongest gains in computational efficiency.

## Methods

### The vectorized non-parametric bootstrap

Let *X* represent a random variable distributed according to an unknown probability distribution *F*, and let ***x*** = (*x*_1_, …, *x*_*N*_)^*t*^ be an observed random sample from *F*. The goal of the bootstrap is to estimate a parameter of interest, *θ*, based on a statistic $\hat{\theta }=s(\text{x})$.

Let $\hat{F}$ represent the empirical distribution of the observed data, ***x***, assigning probability 1/*N* to each observed value *x*_*i*_, *i* = 1, …, *N*. A bootstrap sample, ${\text{x}}^{*}={\left(x_{1}^{*},\ldots ,x_{N}^{*}\right)}^{t}$, corresponds to a random sample of size *N* draw from $\hat{F}$. Operationally, sampling from $\hat{F}$ is equivalent to sampling *N* data points with replacement from the population of *N* objects (*x*_1_, …, *x*_*N*_). The star notation indicates that x* is not the actual data set ***x*** but rather a re-sampled version of ***x***. The sampling distribution of estimator $\hat{\theta }$ is then estimated from *B* bootstrap replications of ${\hat{\theta }}^{*}=s({\text{x}}^{*})$.

Now, consider the estimation of the first moment of the unknown probability distribution *F*,

$$\begin{array}{c} \theta =E_{F}\left(X\right)\;, \end{array}$$ (1)

on the basis of the observed data ***x***. If no further information (other than the observed sample ***x***) is available about *F*, then it follows that the best estimator of *θ* is the plug-in estimate (see page 36 of [2]),

$$\begin{array}{c} \hat{\theta }\;=\;E_{\hat{F}}\left(X\right)\;=\;\sum_{i=1}^{N}x_{i}\;P_{\hat{F}}\left(X=x_{i}\right)\;=\;\sum_{i=1}^{N}x_{i}\frac{1}{N}\;=\;s\left(\chi \right)\;, \end{array}$$ (2)

and the bootstrap distribution of $\hat{\theta }$ is generated from *B* bootstrap replications of ${\hat{\theta }}^{*}=s({\text{x}}^{*})=N^{-1}\sum_{i=1}^{N}x_{i}^{*}$. Algorithm 1 summarizes the approach.

**Algorithm 1** Non-parametric bootstrap for $E_{\hat{F}}(X)$ via data re-sampling

1: *For b* = 1, …, *B*:

- Draw a bootstrap sample ${\text{x}}^{*}={\left(x_{1}^{*},\ldots ,x_{N}^{*}\right)}^{t}$ from the empirical distribution of the observed data, that is, sample *N* data points with replacement from the population of *N* objects ***x*** = (*x*_1_, …, *x*_*N*_)^*t*^.
- Compute the bootstrap replication ${\hat{\theta }}^{*}=N^{-1}\sum_{i=1}^{N}x_{i}^{*}$.

Alternatively, let $n_{i}^{*}$ represent the number of times that data point *x*_*i*_ appears in the bootstrap sample x*, and $w_{i}^{*}=n_{i}^{*}/N$. Then, the category counts, ${\text{n}}^{*}={\left(n_{1}^{*},\ldots ,n_{N}^{*}\right)}^{t}$, of the bootstrap sample x* are distributed according to the multinomial distribution,

$$\begin{array}{c} n^{*}\;=\;N\;w^{*}\;∼\;\text{Multinomial}(N\;,\;N^{-1}\;1_{N})\;, \end{array}$$ (3)

where the vector **1**_*N*_ = (1, …, 1)^*t*^ has length *N*.

Now, since

$$\begin{array}{c} \sum_{i=1}^{N}x_{i}^{*}\;=\;\sum_{i=1}^{N}n_{i}^{*}\;x_{i}\;, \end{array}$$ (4)

it follows that the bootstrap replication of the first sample moment of the observed data can we re-expressed, in terms of the bootstrap weights w* as,

$$\begin{array}{c} {\hat{\theta }}^{*}\;=\;\frac{1}{N}\;\sum_{i=1}^{N}x_{i}^{*}\;=\;\sum_{i=1}^{N}w_{i}^{*}\;x_{i}\;, \end{array}$$ (5)

so that we can generate bootstrap replicates using Algorithm 2.

**Algorithm 2** Non-parametric bootstrap for $E_{\hat{F}}(X)$ via multinomial sampling

1: *For b* = 1, …, *B*:

- Draw a bootstrap count vector n* ˜ Multinomial(*N*, *N* ^−1^ **1**_*N*_).
- Compute the bootstrap weights ${\text{w}}^{*}={\left(n_{1}^{*}/N,\ldots ,n_{N}^{*}/N\right)}^{t}$.
- Compute the bootstrap replication ${\hat{\theta }}^{*}=\sum_{i=1}^{N}w_{i}^{*}\;x_{i}$.

The main advantage of this multinomial sampling formulation of the non-parametric bootstrap is that it allows the vectorization of the computation. Explicitly, Algorithm 2 can be vectorized as follows:

1. Draw *B* bootstrap count vectors, n*, from Eq (3), using a single call of a multinomial random vector generator (e.g., rmultinom in R).
2. Divide the sampled bootstrap count vectors by *N* in order to obtain a *N* × *B* bootstrap weights matrix, W*.
3. Generate the entire vector of bootstrap replications,
  $$\begin{array}{c} {\hat{\theta }}^{*}\;=\;\chi^{t}\;W^{*} \end{array}$$ (6)
  in a single computation.

It is clear from Eq (4) that this multinomial sampling formulation is available for statistics based on any arbitrary sample moment (that is, statistics defined as functions of arbitrary sample moments). For instance, the sample correlation between data vectors ***z*** = (*z*_1_, …, *z*_*N*_)^*t*^ and ***y*** = (*y*_1_, …, *y*_*N*_)^*t*^, is a function of the sample moments,

$$\begin{array}{c} \frac{1}{N}\sum_{i=1}^{N}z_{i}\;,\;\frac{1}{N}\sum_{i=1}^{N}y_{i}\;,\;\frac{1}{N}\sum_{i=1}^{N}z_{i}^{2}\;,\;\frac{1}{N}\sum_{i=1}^{N}y_{i}^{2}\;,\;\frac{1}{N}\sum_{i=1}^{N}z_{i}\;y_{i}\;, \end{array}$$ (7)

and the bootstrap replication,

$$\begin{array}{c} {\hat{\theta }}^{*}\;=\;\frac{N\sum_{i}z_{i}^{*}\;y_{i}^{*}\;-\;\left(\sum_{i}z_{i}^{*}\right)\;\left(\sum_{i}y_{i}^{*}\right)}{\sqrt{\left[N\sum_{i}z_{i}^{*2}-{\left(\sum_{i}z_{i}^{*}\right)}^{2}]\;[N\sum_{i}y_{i}^{*2}-{\left(\sum_{i}y_{i}^{*}\right)}^{2}\right]}}\;, \end{array}$$ (8)

can be re-expressed in terms of bootstrap weights as,

$$\begin{array}{c} {\hat{\theta }}^{*}\;=\;\frac{\sum_{i}w_{i}^{*}\;z_{i}\;y_{i}\;-\;\left(\sum_{i}w_{i}^{*}\;z_{i}\right)\left(\sum_{i}w_{i}^{*}\;y_{i}\right)}{\sqrt{\left[\sum_{i}w_{i}^{*}\;z_{i}^{2}-{\left(\sum_{i}w_{i}^{*}\;z_{i}\right)}^{2}]\;[\sum_{i}w_{i}^{*}\;y_{i}^{2}-{\left(\sum_{i}w_{i}^{*}\;y_{i}\right)}^{2}\right]}}\;, \end{array}$$ (9)

and in vectorized form as,

$$\begin{array}{c} {\hat{\theta }}^{*}\;=\;\frac{{\left(z•y\right)}^{t}W^{*}\;-\;\left(z^{t}W^{*}\right)•\left(y^{t}W^{*}\right)}{\sqrt{\left[{\left(z^{2}\right)}^{t}W^{*}-{\left(zW^{*}\right)}^{2}]•[{\left(y^{2}\right)}^{t}W^{*}-{\left(y^{t}W^{*}\right)}^{2}\right]}}\;, \end{array}$$ (10)

where the • operator represents the Hadamard product of two vectors (that is, the element-wise product of the vectors entries), and the square and square root operations in the denominator of Eq (10) are also performed entry-wise.

## Results

In this section, we illustrate the gain in computational efficiency achieved by the vectorized multinomial sampling bootstrap, relative to three versions of the standard data resampling approach: a straightforward version based on a “for loop”, and two state-of-art R implementations available in the bootstrap [7] and boot [3, 8] R packages. In the following, we refer to these three versions as “for loop”, “R/bootstrap”, and “R/boot”, respectively.

**Fig 1:**
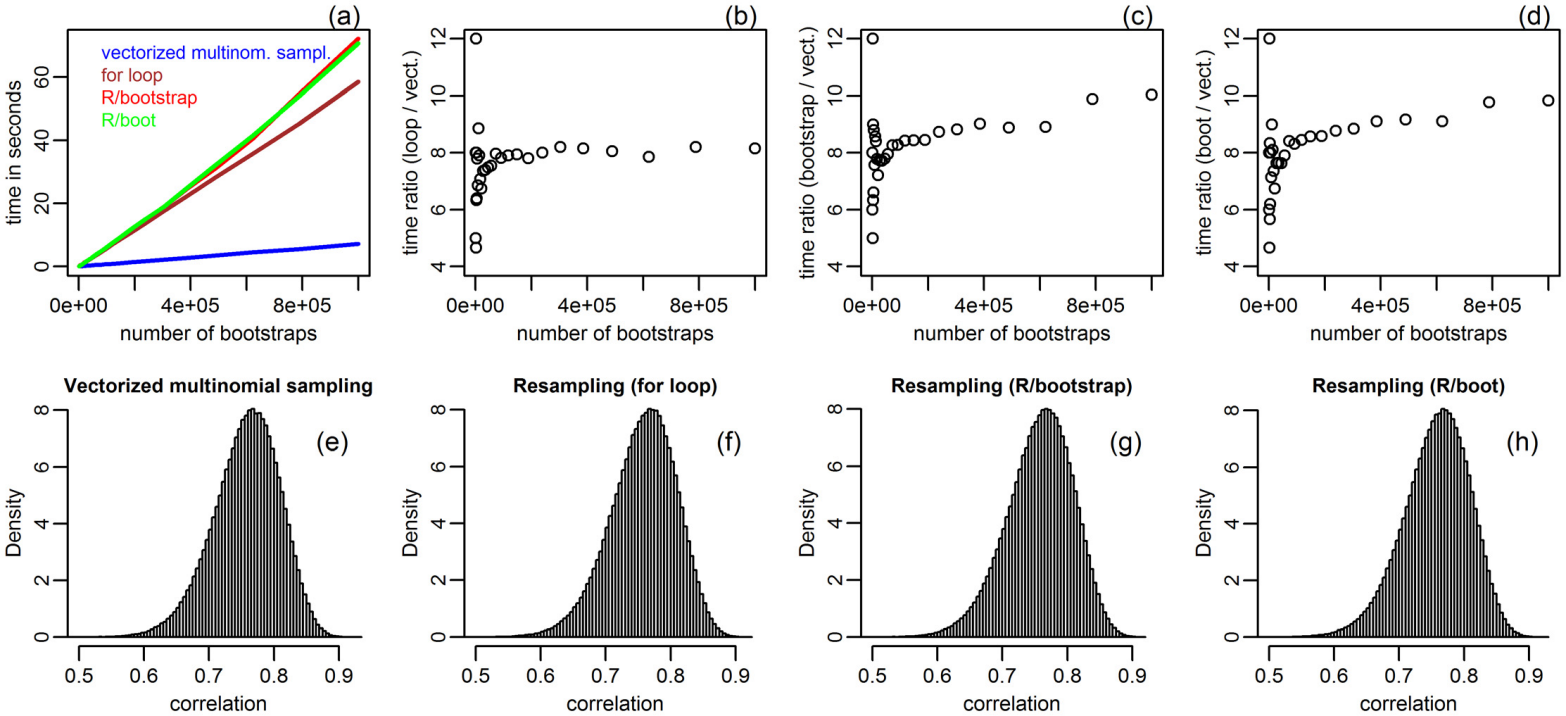
Comparison between the data re-sampling approaches and the vectorized multinomial sampling bootstrap, in the American law school data. Panel a compares the approach’s time expenditures. Panels b, c and d compare, respectively, the computation time ratio of the “for loop”, “R/bootstrap” and “R/boot” approaches against the vectorized bootstrap. The bottom panels present the ${\hat{\theta }}^{*}=\text{c}\hat{\text{o}}\text{r}({\text{LSAT}}^{*},{\text{GPA}}^{*})$ distributions generated with *B* = 1,000,000.

We bootstrapped Pearson’s sample correlation coefficient using the American law school data (page 21 of [2]) provided in the law82 data object of the bootstrap R package. The data is composed of two measurements (class mean score on a national law test, LSAT, and class mean undergraduate grade point average, GPA) on the entering class of 1973 for *N* = 82 American law schools. Fig 1 presents the results.

Fig 1a shows the time (in seconds) required to generate *B* bootstrap replications of ${\hat{\theta }}^{*}=\text{c}\hat{\text{o}}\text{r}({\text{LSAT}}^{*},{\text{GPA}}^{*})$, for *B* varying from 1,000 to 1,000,000. The red, green, brown, and blue lines show, respectively, the computation time required by the “R/bootstrap”, “R/boot”, “for loop”, and the vectorized multinomial sampling approaches. Panels b-d show, respectively, the computation time ratio of the “for loop”, “R/bootstrap” and “R/boot” approaches versus the vectorized bootstrap, as a function of *B*. The plots clearly show that the vectorized bootstrap was considerably faster than the data re-sampling implementations for all *B* tested. The bottom panels show the ${\hat{\theta }}^{*}$ distributions for the four bootstrap approaches based on *B* = 1,000,000.

**Fig 2:**
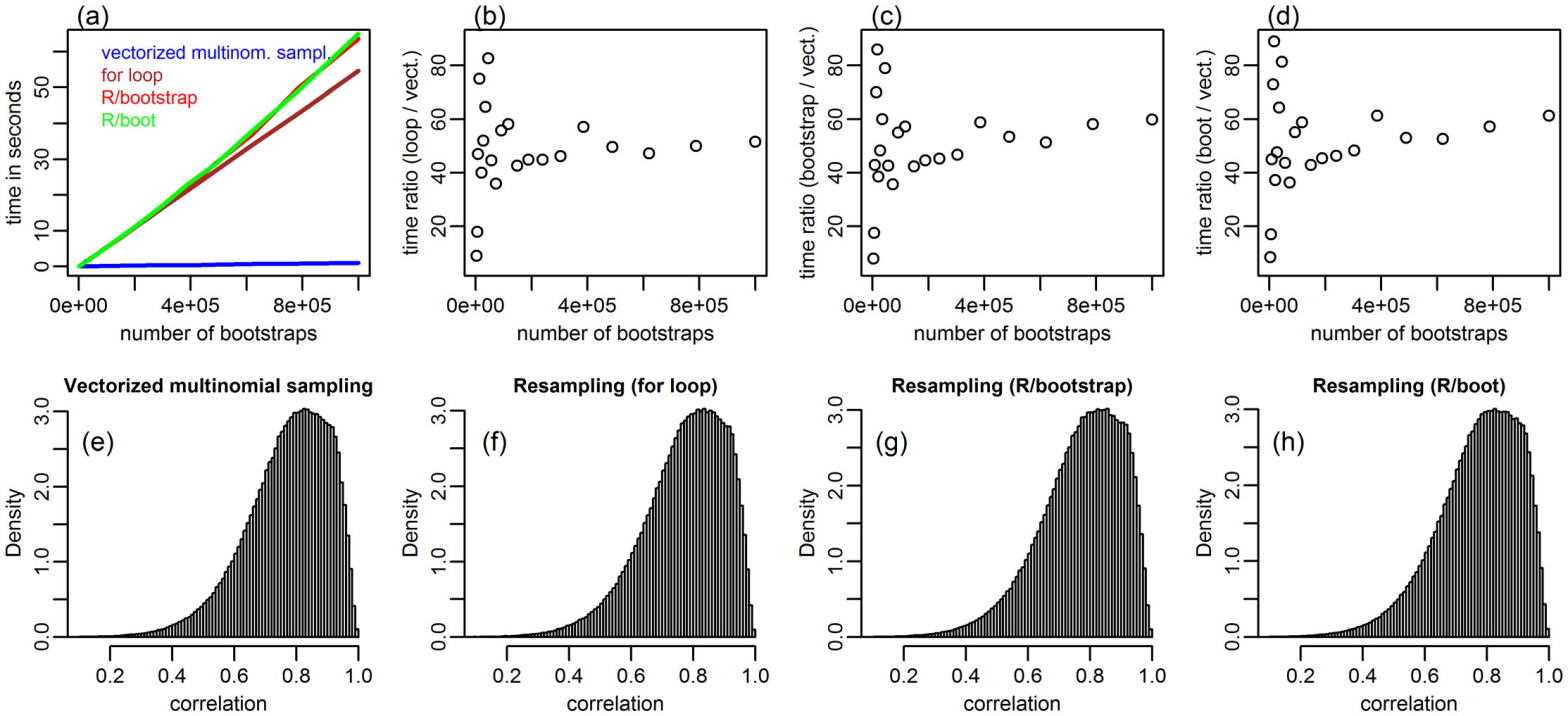
Comparison between the data re-sampling approaches and the vectorized multinomial sampling bootstrap, in a subset of the American law school data. Panel a compares the approach’s time expenditures. Panels b, c and d compare, respectively, the computation time ratio of the “for loop”, “R/bootstrap” and “R/boot” approaches against the vectorized bootstrap. The bottom panels present the ${\hat{\theta }}^{*}=\text{c}\hat{\text{o}}\text{r}({\text{LSAT}}^{*},{\text{GPA}}^{*})$ distributions generated with *B* = 1,000,000.

Fig 2 presents analogous comparisons, but now focusing on a subset of *N* = 15 samples from the American law school data (page 19 of [2]), provided in the law data object of the bootstrap R package. This time, the vectorized implementation was remarkably faster than the data re-sampling versions. Panels b-d of Fig 2 show that the vectorized implementation was roughly 50 times faster than the resampling versions, whereas in the previous example it was about 8 times faster (Fig 1b-1d).

**Fig 3:**
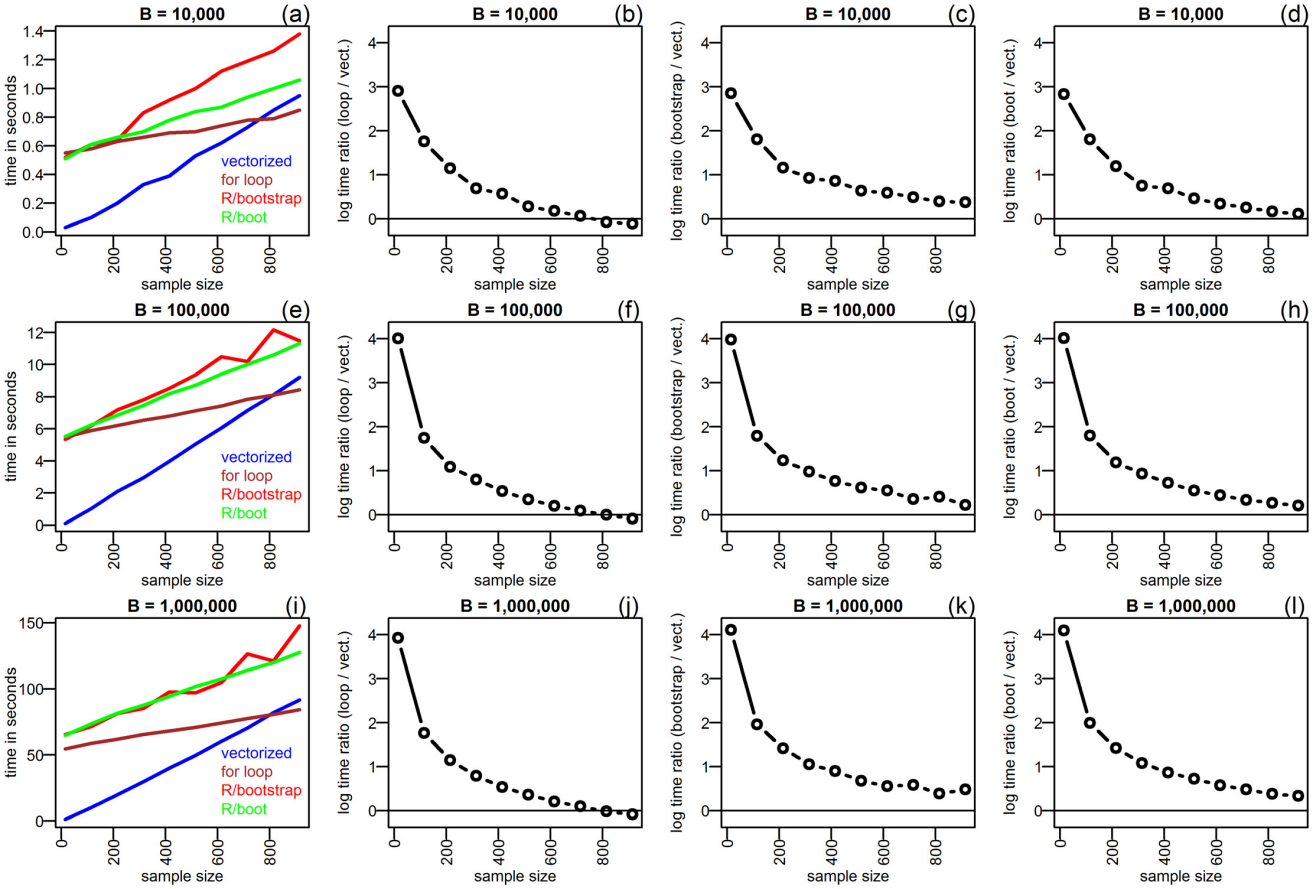
Comparison of the bootstrap implementations, when bootstrapping Pearson’s sample correlation, ${\hat{\theta }}^{*}=\text{c}\hat{\text{o}}\text{r}({\text{x}}_{1}^{*},{\text{x}}_{2}^{*})$, for sample sizes varying from 15 to 915. The data was simulated according to *x*_1*i*_ = *ϵ*_1*i*_, *x*_2*i*_ = *x*_1*i*_+*ϵ*_2*i*_, *ϵ*_*ji*_ ∼ N(0,1), *j* = 1,2. Results based on 10,000 (panels a-d), 100,000 (panels e-h), and 1,000,000 (panels i-l) bootstrap replications. The left panels (a, e, and i) compare the approach’s time expenditures. The remaining panels show the computation time ratios (in log scale) comparing the resampling approaches against the vectorized implementation. The horizontal line is set at zero and represents the threshold below which the data resampling approach outperforms the vectorized implementation.

The performance difference observed in these two examples suggests that the gain in speed achieved by the vectorized implementation decreases as a function of the sample size. In order to confirm this observation, we used simulated data to compare the bootstrap implementations across a grid of 10 distinct sample sizes (varying from 15 to 915) for *B* equal to 10,000, 100,000, and 1,000,000. Fig 3 reports the results.

The left panels (a, e, and i) of Fig 3 show computation times as a function of increasing sample sizes for all four approaches. In all cases tested the vectorized implementation (blue line) outperformed the “R/bootstrap” (red line) and the “R/boot” (green line), whereas the “for loop” version (brown line) outperformed the vectorized implementation for large sample sizes (but was considerably slower for small and moderate sample sizes). Panels b, f, and j show the computation time ratios (in log scale) comparing the “for loop” vs vectorized implementations, whereas panels c, g, and k compare the “R/bootstrap” vs vectorized, and panels d, h, and l compare the “R/boot” vs vectorized. The horizontal line is set at zero and represents the threshold below which the data resampling approach outperforms the vectorized implementation. Note that log time ratios equal to 4, 3, 2, 1, and 0.5, correspond to speed gains of 54.60, 20.09, 7.39, 2.72, and 1.65 seconds, respectively.

**Fig 4:**
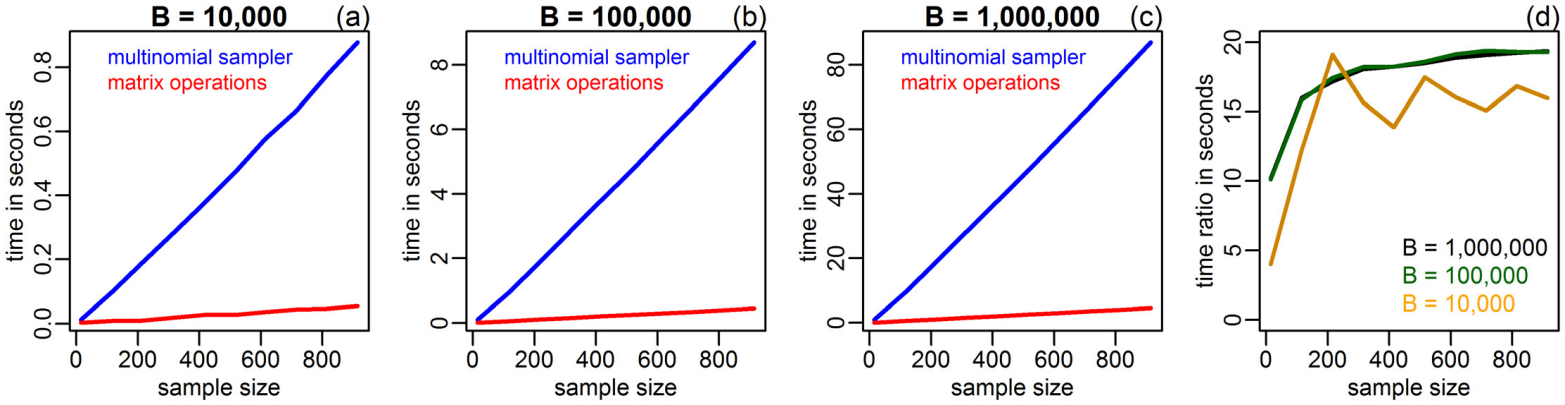
Comparison of the time spent in the generation of the bootstrap weights matrix, W*, versus the time spent on all other matrix/vector operations involved in the calculation of the vector of bootstrap replications. Panel a reports the time (in seconds) spent in the generation of W* (blue curve) and on the remaining matrix operations (red curve) as a function of sample sizes varying from 15 to 915, when *B* = 10,000. Panels b and c present analogous results for *B* equal to 100,000, and 1,000,000, respectively. Panel d presents the ratio of the time spent in the generation of W* against all other matrix/vector operations, for *B* equal to 10,000, 100,000, and 1,000,000.

In order to further clarify why the gain in speed achieved by the vectorized bootstrap decreases as a function of the sample size, we compared the time spent in the generation of the bootstrap weights matrix, W* (via multinomial sampling with the rmultinom R base function), against the time expenditure on all other matrix/vector operations involved in the calculation of the vector of bootstrap replications. Fig 4 presents the results. Panels a-c report the time (in seconds) spent in the generation of W* (blue curve) and on the remaining matrix operations (red curve) against sample sizes, *N*, varying from 15 to 915, for *B* equal to 10,000, 100,000, and 1,000,000. These panels suggest that computation time scales linearly with both sample size and number of bootstrap replications (note the ten fold increase in computation times reported in the y-axis of panels a-c, as a function of 10 fold increments in *B*), and clearly show that the bulk of the computation time is allocated to the generation of the bootstrap weights matrix. Panel d further illustrates this last point by presenting the ratio of the time spent in the generation of W* against all other matrix/vector operations.

All the timings in this section were measured on an Intel Core i7-3610QM (2.3 GHz), 24 Gb RAM, Windows 7 Enterprize (64-bit) platform. All the R code used in the generation of these results is available at https://github.com/echaibub/VectorizedNonParametricBootstrap.

## Discussion

In this paper we showed how the multinomial sampling formulation of the non-parametric bootstrap can be easily implemented in vectorized form. We illustrate the gain in computational speed (in the R programming language) using real and simulated data sets.

Our examples provide several interesting insights. First, the resampling implementation based on the “for loop” was generally faster than the implementations provided by the bootstrap and boot R packages. Panels a, e, and i of Fig 3 further show that the speed difference between the “for loop” and the “R/bootstrap” implementations gets larger as a function of increasing sample sizes (increasing gap between the brown and red curves). Comparison of the “for loop” and the “R/boot” implementations shows a similar pattern (increasing gap between the brown and green curves). These observations might be partially explained by the fact that our straightforward “for loop” implementation does not engage in the same extensive data checks required by the bootstrap and boot packages, in order to ensure code stability/robustness and proper execution of the additional functionality implemented in these packages. Furthermore, the bootstrap package employs a apply call in place of the for loop, which might also contribute to the speed gap, since the apply call can be slower than a for loop when handling large objects (see [9] for further discussion and examples). Second, the vectorized bootstrap outperformed the data resampling implementations provided in the bootstrap and boot R packages in all cases tested (compare the blue, red and green curves in panel a of Figs 1 and 2, and on the left panels of Fig 3). Third, the gain in speed achieved by the vectorized implementation decreases as a function of the sample size (Fig 3). As shown in Fig 4, this decrease is mostly explained by the increase in the time required for sampling a *N* × *B* matrix of multinomial counts, using the rmultinom function. We point out, however, that even though the vectorized implementation was slightly slower than the simple “for loop” approach for large *N*, our examples still show remarkable/considerable gains for small/moderate sample sizes, in comparison to all three data resampling approaches. Fourth, from a practical point of view, the use of the vectorized implementation is straightforward. Similarly, to the bootstrap and boot R packages, the user only needs to specify a R function evaluating the statistic of interest (in vectorized format). S1 Text provides a R tutorial with a few illustrative examples.

All the results presented in the previous section were generated using single thread computations, without leveraging any of the standard parallelization capabilities provided in R. As any other bootstrap technique, the vectorized implementation can potentially benefit from parallelization by splitting and distributing the total number of bootstrap calculations across multiple cores. In order to investigate the relative performances of parallelized versions of the “for loop” and vectorized approaches (exploring the functionality provided by the parallel R package [5]), we compared the running times of the single threaded versus parallel implementations in all the real and simulated data comparisons presented in this article. Our main findings included that: (i) similarly to the results observed in the single thread computations, the vectorized parallel implementations still tended to out-perform the “for loop” parallel implementations in small and/or moderate sample sizes, but tended to be slower for larger sample sizes; (ii) for larger sample sizes and larger number of bootstrap replications, the parallel implementations tended to outperform the single threaded computations, although for small sample sizes/bootstrap replications the single thread approaches were at times faster (illustrating that, sometimes, the time spent in distributing and gathering results across multiple cores can be greater than the time needed for carrying out the single thread computations). Detailed descriptions of the parallel implementations, as well as, of the benchmarking study, are presented on S2 Text. We point out, nonetheless, that benchmarking results for parallel implementations are highly dependent on hardware specifications.

For the sake of clarity, the exposition in the Methods section focused on statistics based on sample moments of the observed data, ***x***. We point out, however, that the multinomial sampling formulation of the bootstrap is available for any statistic satisfying the more general relation,

$$\begin{array}{c} \sum_{i=1}^{N}\;f\left(x_{i}^{*}\right)\;=\;\sum_{i=1}^{N}\;n_{i}^{*}\;f\left(x_{i}\right)\;, \end{array}$$ (11)

where *f*(*x*_*i*_) represents a transformation of the original data. Note, nonetheless, that the left hand side of Eq (11) still represents a bootstrap replication of the first sample moment of the transformed variable *u*_*i*_ = *Nf*(*x*_*i*_), and that the *k*th sample moment represents the particular case, $u_{i}=x_{i}^{k}$.

The multinomial sampling formulation of the non-parametric bootstrap is not new. It is actually a key piece in the demonstration of the connection between the non-parametric bootstrap and Bayesian inference, described in [10] and in Section 8.4 of [11], where the non-parametric bootstrap is shown to closely approximate the posterior distribution of the quantity of interest generated by the Bayesian bootstrap [10]. This close connection, also implies that the Bayesian bootstrap can be easily implemented in vectorized form. As a matter of fact, instead of generating the bootstrap weights from bootstrap count vectors sampled from a multinomial distribution, the Bayesian bootstrap samples the weights directly from a Dirichlet(**1**_*N*_) distribution. We point out, however, that, to the best of our knowledge, the multinomial sampling formulation has not been explored before for vectorizing bootstrap computations.

Another interesting approach, which can also be easily vectorized, is the approximate non-parametric bootstrap based on Poisson frequencies [12]. The basic idea is that, instead of sampling the bootstrap frequencies, ${\text{n}}^{*}={\left(n_{1}^{*},\ldots ,n_{N}^{*}\right)}^{t}$, from a Multinomial(*N*, *N* ^−1^ **1**_*N*_) distribution, one can sample *N* random frequencies, $\left(p_{1}^{*},\ldots ,p_{N}^{*}\right)$, from a Poisson distribution with expectation 1. Note that whereas in the multinomial version the sum of the bootstrap frequencies, $\sum_{i=1}^{N}n_{i}^{*}$, is always *N*, the Poisson frequencies do not sum to *N*, although the expectation of $\sum_{i=1}^{N}p_{i}^{*}$ is *N*. In other words, the multinomial bootstrap generates samples of fixed size *N*, while the Poisson approach generates samples with variable sizes (but equal to *N*, on average). For instance, when bootstrapping the mean, $\bar{y}=N^{-1}\sum_{i=1}^{N}y_{i}$, the multinomial approach uses ${\bar{y}}^{*}=N^{-1}\sum_{i=1}^{N}n_{i}^{*}y_{i}$, whereas the Poisson frequencies approach uses ${\bar{y}}^{*}=(\sum_{i=1}^{N}p_{i}^{*}y_{i})/(\sum_{i=1}^{N}p_{i}^{*})$. In order to check whether sampling from a Poisson distribution could lead to a computationally more efficient vectorized bootstrap (and also evaluate the quality of the approximation) we benchmarked the Poisson approach against the multinomial sampling bootstrap, using the American law schools data. Our results show, nonetheless, that the multinomial sampling approach tends to be faster (see S3 Text for details).

Finally, we point out that even though the vectorized bootstrap is straightforward from a statistical/mathematical standpoint, it still represents an important contribution to the scientific community, since moment based statistics are widely used in practice and the speed improvements reported in this paper will likely be the rule and not the exception (as experiments based on small/moderate sample sizes are usually the norm in the majority of scientific fields). The gain in computational efficiency achieved by our vectorized implementation might encourage users to adopt larger numbers of bootstrap replications, allowing for more precise estimation of bootstrap p-values, bootstrap confidence intervals, and the statistic’s sampling distribution. Furthermore, the vectorized non-parametric bootstrap might be most appealing when the user needs to apply the bootstrap to a large number of small and parallel experiments, since the speed improvements of each separate application can quickly add up to represent the difference between a few hours versus several days of computation.

## Supporting Information

S1 Text Vectorized bootstrap R tutorial. This file contains a tutorial illustrating the use of vectorized R code for the non-parametric bootstrap. The companion R code implementing the vectorized non-parametric bootstrap, as well as, the script used to generate this tutorial and (all the results presented in this article) is available at https://github.com/echaibub/VectorizedNonParametricBootstrap.
(PDF)

S2 Text Comparison of single thread versus parallel implementations. This file presents a benchmark study comparing single thread “for loop” and vectorized implementations, against parallel “for loop” and vectorized implementations (based on both the parLapply and mclapply alternatives to the lapply function, provided by the parallel R package).
(PDF)

S3 Text Comparison of the bootstraps based on multinomial and Poisson frequencies. This file presents a benchmarking study comparing the vectorized bootstrap based on multinomial frequencies against a vectorized implementation of the approximate bootstrap based on Poisson frequencies.
(PDF)
