# Supplementary material for: Speeding Up Non-Parametric Bootstrap Computations for Statistics Based on Sample Moments in Small/Moderate Sample Size Applications
Source: PLoS One. 2015 Jun 30;10(6):e0131333. doi: 10.1371/journal.pone.0131333 (PMC4488363; doi:10.1371/journal.pone.0131333)
Supplement: S1 Text — This file contains a tutorial illustrating the use of vectorized R code for the non-parametric bootstrap. The companion R code implementing the vectorized non-parametric bootstrap, as well as, the script used to generate this tutorial and (all the results presented in this article) is available at https://github.com/echaibub/VectorizedNonParametricBootstrap. (PDF) [file pone.0131333.s001.pdf]

S1 Text. Vectorized bootstrap R tutorial

In practice, the use of vectorized implementations of the non-parametric bootstrap is straightforward. Similarly to the `boot` [1,2] and `bootstrap` [3] R packages, the user only needs to specify an R function for computing the statistic of interest in vectorized format. In the following we illustrate this point with a few examples.

First we start by sourcing the code implementing the basic vectorized bootstrap functions from the "VectorizedNonParametricBootstrap" github repository.

```
> library(devtools)
> codeUrl <- paste("https://raw.githubusercontent.com", "echaibub",
+                  "VectorizedNonParametricBootstrap", "master",
+                  "vectorized_bootstrap_functions.R", sep = "/")
> source_url(codeUrl)
```

Next, we load the American law schools data [2] available from the `bootstrap` R package. The `law` object contains a subset ($N = 15$) of the American law schools data, which is composed of two variables (class mean score on a national law test, LSAT, and class mean undergraduate grade point average, GPA).

```
> data(law)
> N <- nrow(law)
> x1 <- law$LSAT
> x2 <- law$GPA
> x <- cbind(x1, x2)
```

As a first example, lets consider the LSAT measurements (assigned to the `x1` variable), and let's bootstrap the sample average statistic,

$$\bar{x}_1 = \frac{1}{N} \sum_{i=1}^{N} x_{1i} \ . \tag{1}$$

A simple function, for bootstrapping this statistic (via data re-sampling) using a "for loop" is given by,

```
LoopMeanBootstrap <- function(B, x) {
  N <- length(x)
  out <- rep(NA, B)
  for (i in seq(B)) {
    idx <- sample(N, replace = TRUE)
    out[i] <- mean(x[idx])
  }
  out
}
```

where the inputs B and x represent, respectively, the number of bootstrap replications and the original data vector. The function's output is a vector of length B with the bootstrap replications,

$$\bar{x}_1^* = \frac{1}{N} \sum_{i=1}^{N} x_{1i}^* \ , \tag{2}$$

of the $\bar{x}_1$ statistic.

Now, since

$$\sum_{i=1}^{N} x_{1i}^* = \sum_{i=1}^{N} n_i^* \, x_{1i} \ , \tag{3}$$

where $\boldsymbol{n}^* = (n_1^*, \ldots, n_N^*)^t$ corresponds to draw from a Multinomial $\big(N\,,\, N^{-1}\,\mathbf{1}_N\big)$ distribution, it follows that $\bar{x}_1^*$ can we re-expressed, in terms of the bootstrap weights $\boldsymbol{w}^* = \boldsymbol{n}^*/N$ as,

$$\bar{x}_1^* \;=\; \frac{1}{N}\sum_{i=1}^{N} x_{1i}^* \;=\; \frac{1}{N}\sum_{i=1}^{N} n_i^*\, x_{1i} \;=\; \sum_{i=1}^{N} w_i^*\, x_{1i} \;=\; \boldsymbol{x}_1^t \boldsymbol{w}^* \;, \tag{4}$$

where $\boldsymbol{x}_1 = (x_{11}, x_{12}, \ldots, x_{1N})^t$. Now, if we catenate B independent multinomial weight vectors into a single $N \times B$ weight matrix, $\boldsymbol{W}^*$, we can compute the entire vector of bootstrap replications into a single matrix multiplication operation,

$$\bar{\boldsymbol{x}}_1^* \;=\; \boldsymbol{x}_1^t \boldsymbol{W}^* \;, \tag{5}$$

with $\bar{\boldsymbol{x}}_1^* = (\bar{x}_{11}^*, \bar{x}_{12}^*, \ldots, \bar{x}_{1B}^*)$.

The vectorized non-parametric bootstrap is implemented in the function,

```
VectorizedBootstrap <- function(N, B, theta, ...) {
  call <- match.call()
  W <- BootWeights(N, B)
  theta.star <- theta(W, ...)
  as.vector(theta.star)
}
```

where the inputs `N` and `B` represent, respectively, the sample size and the number of bootstrap replications. The `theta` argument represents a function, specified by the user, which computes the statistic of interest in vectorized format. For instance, in our example with the $\bar{x}_1^*$ statistic, a vectorized function is given by,

```
SampleBootMean <- function(W, x) {
  crossprod(x, W)
}
```

where `W` represents a $N \times B$ matrix of multinomial weights generated internally in the `VectorizedBootstrap` function by a call to,

```
BootWeights <- function(N, B) {
  counts <- rmultinom(B, N, rep(1/N, N))
  counts/N
}
```

The `...` argument in the `VectorizedBootstrap` function, represents additional arguments to be passed the `theta` function, in addition to the multinomial weights matrix `W`. For example, in order to generate 1,000 bootstrap replications of the $\bar{x}_1^*$ statistic we would use `x1` as the additional argument to be passed to `SampleBootMean` with the call,

```
VectorizedBootstrap(N = length(x1), B = 1e+3, theta = SampleBootMean, x1)
```

As an illustration we now generate 1e+5 bootstrap replications of $\bar{x}_1^*$, using the "for loop" and the vectorized implementations, as well as, the `R/boot` and `R/bootstrap` packages.

```
> B <- 1e+5
> set.seed(123)
> system.time( b1 <- LoopMeanBootstrap(B, x1) )
   user  system elapsed
   1.56    0.00    1.59
> system.time( b2 <- VectorizedBootstrap(N, B, SampleBootMean, x1) )
```

```
   user  system elapsed
   0.10    0.00    0.09
> system.time( b3 <- boot(x1, ThetaMean2, B, stype = "i")$t[, 1] )
   user  system elapsed
   1.31    0.00    1.33
> system.time( b4 <- bootstrap(seq(N), B, ThetaMean, x1)$thetastar )
   user  system elapsed
   1.34    0.03    1.38
```

The functions `ThetaMean <- function(idx, x) {mean(x[idx])}` and `ThetaMean2 <- function(x, idx) {mean(x[idx])}` in the `bootstrap` and `boot` calls, compute the sample mean statistic on re-sampled versions of the data. The time (in seconds) taken by each of these four bootstrap implementations is reported by the `system.time` function. Next, we plot histograms of the bootstrap distributions.

```
> par(mfrow = c(2, 2))
> hist(b1, nclass = 50, main = "for loop", xlab = "mean")
> hist(b2, nclass = 50, main = "vectorized bootstrap", xlab = "mean")
> hist(b3, nclass = 50, main = "R/boot", xlab = "mean")
> hist(b4, nclass = 50, main = "R/bootstrap", xlab = "mean")
> par(mfrow = c(1, 1))
```

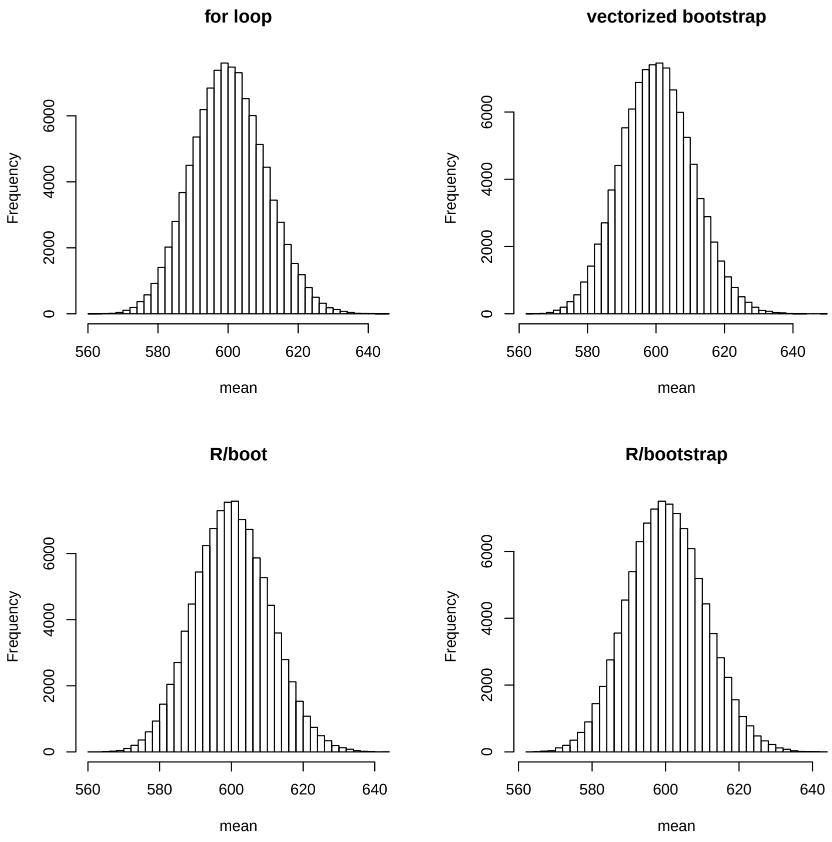

Our next example, shows how to bootstrap Pearson's sample correlation coefficient in vectorized format. In this case, the straightforward "for loop" implementation is given by,

```
LoopCorBootstrap <- function(B, x1, x2) {
  N <- length(x1)
  out <- rep(NA, B)
  for (i in seq(B)) {
    idx <- sample(N, replace = TRUE)
    out[i] <- cor(x1[idx], x2[idx])
  }
  out
}
```

the `R/bootstrap` and `R/boot` functions are given, respectively, by,

```
ThetaCor <- function(idx, x1, x2) {
  cor(x1[idx], x2[idx])
}
ThetaCor2 <- function(x, idx) {
  cor(x[idx, 1], x[idx, 2])
}
```

while the corresponding Pearson's correlation function, in vectorized format, is given by,

```
SampleBootCor <- function(W, x1, x2) {
  xbar.1 <- crossprod(x1, W)
  xbar.2 <- crossprod(x2, W)
  s2.1 <- x1^2
  s2.1 <- crossprod(s2.1, W)
  s2.1 <- s2.1 - xbar.1^2
  s2.2 <- x2^2
  s2.2 <- crossprod(s2.2, W)
  s2.2 <- s2.2 - xbar.2^2
  s.12 <- x1 * x2
  s.12 <- crossprod(s.12, W)
  s.12 <- s.12 - xbar.1 * xbar.2
  s.12/sqrt(s2.1 * s2.2)
}
```

Next, we generate and benchmark the time taken by these four bootstrap implementations to generate 100,000 bootstrap replications of Pearson's sample correlation coefficient.

```
> B <- 1e+5
> set.seed(123)
> system.time( b1 <- LoopCorBootstrap(B, x1, x2) )
   user  system elapsed
   5.59    0.00    5.64
> system.time( b2 <- VectorizedBootstrap(N, B, SampleBootCor, x1, x2) )
   user  system elapsed
   0.11    0.00    0.11
> system.time( b3 <- boot(x, ThetaCor2, B, stype = "i")$t[, 1] )
   user  system elapsed
```

```
   5.71    0.00    5.71
> system.time( b4 <- bootstrap(seq(N), B, ThetaCor, x1, x2)$thetastar )
   user  system elapsed
   5.50    0.00    5.51
```

Next, we plot histograms of the bootstrap distributions.

```
> par(mfrow = c(2, 2))
> hist(b1, nclass = 50, xlim = c(0, 1), main = "for loop", xlab = "correlation")
> hist(b2, nclass = 50, xlim = c(0, 1), main = "vectorized bootstrap",
+      xlab = "correlation")
> hist(b3, nclass = 50, xlim = c(0, 1), main = "R/boot", xlab = "correlation")
> hist(b4, nclass = 50, xlim = c(0, 1), main = "R/bootstrap", xlab = "correlation")
> par(mfrow = c(1, 1))
```

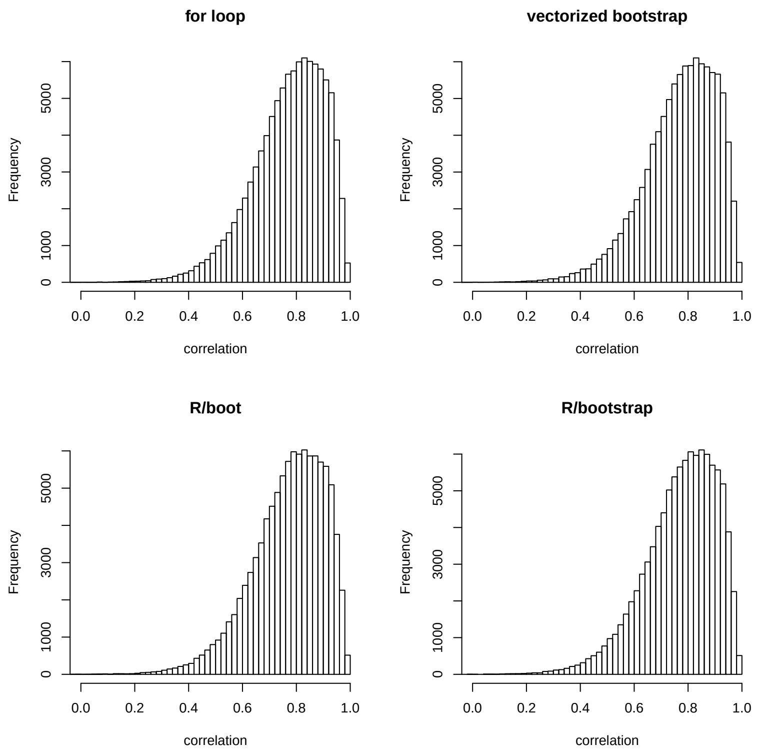

Finally, in our third example we illustrate how to implement the vectorized bootstrap for testing for the equality of means of two populations using Welch's t-test test statistic. For this example, we make use of the mouse data (Table 15.1 on page 206 of [4]) available from the `bootstrap` package. The data corresponds to a small randomized experiment performed on 16 mouse (7 on the treatment group and 9 on the control group), where the treatment was intended to prolong survival after a test surgery. The measurement unit is days of survival following the test surgery.

Our first step is to load the mouse data, and create a data frame (`mouse`) with the days of survival data in the first column and the group codes (1 for treatment, and 2 for control) in the second column

(since application of the `boot` function to multi-sample problems requires we organize the data in this format).

```
> data(mouse.t) ## treatment
> data(mouse.c) ## control
> groups <- c(rep(1, length(mouse.t)), rep(2, length(mouse.c)))
> days <- c(mouse.t, mouse.c)
> mouse <- data.frame(days, groups)
```

In our implementations we follow the algorithm described on page 224 of [4]. Basically, given data on two groups, x_{1i}, $i = 1, \ldots, N_1$ and x_{2i}, $i = 1, \ldots, N_2$, we:

1. Generate transformed data $\tilde{x}_{1i} = x_{1i} - \bar{x}_1 + \bar{x}$ and $\tilde{x}_{2i} = x_{2i} - \bar{x}_2 + \bar{x}$, where $\bar{x}_1$ and $\bar{x}_2$ are the groups means and $\bar{x}$ is the mean of the combined sample.

2. Create B bootstrap data sets $(\boldsymbol{x}_1^*, \boldsymbol{x}_2^*)$ with $\boldsymbol{x}_1^*$ sampled with replacement from $\{\tilde{x}_{1i},\, i = 1, \ldots, N_1\}$ and $\boldsymbol{x}_2^*$ sampled with replacement from $\{\tilde{x}_{2i},\, i = 1, \ldots, N_2\}$.

3. Evaluate Welch's statistic,
$$\frac{\bar{x}_1^* - \bar{x}_2^*}{\sqrt{s_1^2/N_1 + s_2^2/N_2}} \;,$$
on each bootstrap data set.

The straightforward "for loop" implementation is given by,

```
LoopWelchsBootstrap <- function(B, x1, x2) {
  n1 <- length(x1)
  n2 <- length(x2)
  n <- n1 + n2
  stat <- rep(NA, B)
  x.bar <- mean(c(x1, x2))
  x1.tilda <- x1 - mean(x1) + x.bar
  x2.tilda <- x2 - mean(x2) + x.bar
  for (i in seq(B)) {
    i1 <- sample(n1, replace = TRUE)
    i2 <- sample(n2, replace = TRUE)
    stat[i] <- WelchsTestStat(x1.tilda[i1], x2.tilda[i2])
  }
  stat
}
```

where the function,

```
WelchsTestStat <- function(x1, x2) {
  n1 <- length(x1)
  n2 <- length(x2)
  s2.1 <- var(x1)
  s2.2 <- var(x2)
  xbar.1 <- mean(x1)
  xbar.2 <- mean(x2)
  s.12 <- sqrt((s2.1/n1) + (s2.2/n2))
  (xbar.1 - xbar.2)/s.12
}
```

computes Welch's test statistic. Our vectorized implementation is given by,

```
SampleBootWelchs <- function(W1, W2, x1, x2) {
  n1 <- length(x1)
  n2 <- length(x2)
  x.bar <- mean(c(x1, x2))
  x1.tilda <- x1 - mean(x1) + x.bar
  x2.tilda <- x2 - mean(x2) + x.bar
  xbar.1 <- crossprod(x1.tilda, W1)
  xbar.2 <- crossprod(x2.tilda, W2)
  s2.1 <- x1.tilda^2
  s2.1 <- crossprod(s2.1, W1)
  s2.1 <- s2.1 - xbar.1^2
  s2.1 <- n1 * s2.1/(n1 - 1)
  s2.2 <- x2.tilda^2
  s2.2 <- crossprod(s2.2, W2)
  s2.2 <- s2.2 - xbar.2^2
  s2.2 <- n2 * s2.2/(n2 - 1)
  s.12 <- sqrt((s2.1/n1) + (s2.2/n2))
  (xbar.1 - xbar.2)/s.12
}
```

The `R/bootstrap` package is unable to handle two-sample problems directly, and for this reason we don't use it in this third example (it requires that the user fit separate bootstraps to the treatment and control groups data in order to generate bootstrap replication vectors for the means and variances of each group separately, and then combine the results externally to generate the bootstrap replications of Welch's statistic).

The `R/boot` package handles multi-sample problems by using an extra argument, "strata", with the groups codes, and by performing stratified re-sampling on the specified strata. The user also needs to specify the statistic function in terms of the data and a vector of frequencies (`stype = "f"`), instead of a vector of indices (`stype = "i"`), which we used in our previous examples. The function for computing Welch's statistic in terms of frequencies is given by,

```
ThetaWelchs <- function(x, f) {
  i1 <- which(x[, 2] == 1)
  i2 <- which(x[, 2] == 2)
  n1 <- length(i1)
  n2 <- length(i2)
  x.tilda <- x
  x.tilda[i1, 1] <- x[i1, 1] - mean(x[i1, 1]) + mean(x[, 1])
  x.tilda[i2, 1] <- x[i2, 1] - mean(x[i2, 1]) + mean(x[, 1])
  x <- x.tilda
  xbar.1 <- sum(x[i1, 1] * f[i1])/sum(f[i1])
  s2.1 <- sum(x[i1, 1]^2 * f[i1])/sum(f[i1]) - xbar.1^2
  s2.1 <- n1 * s2.1/(n1 - 1)
  xbar.2 <- sum(x[i2, 1] * f[i2])/sum(f[i2])
  s2.2 <- sum(x[i2, 1]^2 * f[i2])/sum(f[i2]) - xbar.2^2
  s2.2 <- n2 * s2.2/(n2 - 1)
  s.12 <- sqrt((s2.1/n1) + (s2.2/n2))
  (xbar.1 - xbar.2)/s.12
}
```

Next, we generate and benchmark the time taken by these three bootstrap implementations to generate 100,000 bootstrap replications of Welch's statistic.

```
> B <- 1e+5
> set.seed(123)
> system.time( b1 <- LoopWelchsBootstrap(B, x1 = mouse.t, x2 = mouse.c) )
   user  system elapsed
   7.18    0.02    7.32
> system.time( b2 <- Vectorized2SampleBootstrap(length(mouse.t),
+                                                length(mouse.c),
+                                                B, SampleBootWelchs,
+                                                mouse.t, mouse.c) )
   user  system elapsed
   0.11    0.00    0.10
> system.time( b3 <- boot(mouse, ThetaWelchs, R = B, stype = "f",
+                          strata = mouse[, 2])$t[, 1] )
   user  system elapsed
  40.11    0.00   40.11
```

Note that we call a two sample version of the `VectorizedBootstrap` function,

```
Vectorized2SampleBootstrap <- function(N1, N2, B, theta, ...) {
  call <- match.call()
  W1 <- BootWeights(N1, B)
  W2 <- BootWeights(N2, B)
  theta.star <- theta(W1, W2, ...)
  as.vector(theta.star)
}
```

which generates separate multinomial weight matrices for the separate groups. For multi-sample problems the user needs to modify the above function to generate the required number of multinomial weight matrices. Next, we plot histograms of the bootstrap distributions.

```
> par(mfrow = c(1, 3))
> hist(b1, nclass = 100, probability = TRUE, xlim = c(-7, 7),
+      main = "for loop", xlab = "Welch's t statistic")
> hist(b2, nclass = 100, probability = TRUE, xlim = c(-7, 7),
+      main = "vectorized", xlab = "Welch's t statistic")
> hist(b3, nclass = 100, probability = TRUE, xlim = c(-7, 7),
+      main = "R/boot", xlab = "Welch's t statistic")
> par(mfrow = c(1, 1))
```

Since the treatment was intended to prolong survival after a surgery, we want to perform a one tailed hypothesis test with the alternative hypothesis that survival is longer in the treatment than in the control group. The corresponding bootstrap p-values are given by,

```
> obs <- WelchsTestStat(x1 = mouse.t, x2 = mouse.c)
> obs
[1] 1.059062
> sum(b1 >= obs)/B
[1] 0.14529
> sum(b2 >= obs)/B
```

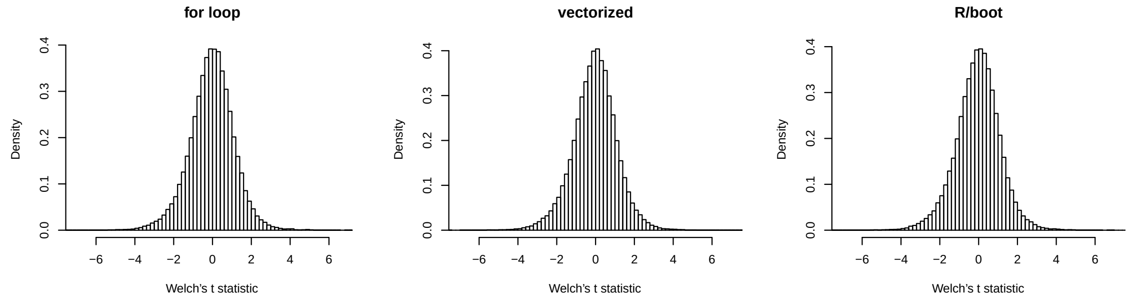


```
[1] 0.14319
> sum(b3 >= obs)/B
[1] 0.14438
```

Note how close the bootstrap p-values are. For the sake of comparison, we also perform an analytical Welch's t-test on the mouse data.

```
> tt <- t.test(mouse.t, mouse.c, var.equal = FALSE, alternative = "greater")
> tt
Welch Two Sample t-test
data:  mouse.t and mouse.c
t = 1.0591, df = 9.645, p-value = 0.1577
alternative hypothesis: true difference in means is greater than 0
95 percent confidence interval:
 -21.98945       Inf
sample estimates:
mean of x mean of y
 86.85714  56.22222
```

We see that the analytical p-value is slightly larger than the bootstrap p-values. Next, we generate 1,000,000 bootstrap replications (what can be done quickly using the vectorized implementation) and compare the bootstrap distribution to the analytical null distribution (blue curve) of Welch's t-test. The value of the observed test statistic is shown by the red vertical line.

```
> set.seed(123)
> system.time( bb2 <- Vectorized2SampleBootstrap(length(mouse.t), length(mouse.c),
+                                                1e+6, SampleBootWelchs, mouse.t,
+                                                mouse.c) )
   user  system elapsed
   1.26    0.04    1.31
> sum(bb2 >= obs)/1e+6
[1] 0.144913
> xaxis <- seq(-6, 6, length.out = 1000)
> densi <- dt(xaxis, tt$parameter)
> hist(bb2, nclass = 300, probability = TRUE, xlim = c(-6, 6),
+      main = "vectorized", xlab = "Welch's statistic")
> lines(xaxis, densi, col = "blue", lwd = 2)
> abline(v = obs, col = "red", lwd = 2)
```

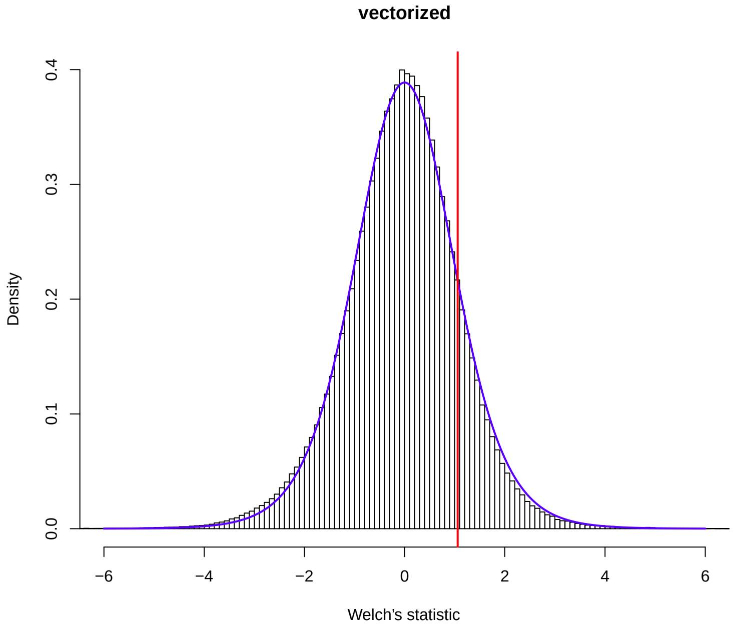

An R script with all the code presented in this tutorial is available at:

`https://raw.githubusercontent.com/echaibub/VectorizedNonParametricBootstrap/master/tutorial_script.R`

References

1. Canty A, Ripley B. boot: Bootstrap R (S-Plus) Functions. R package version 1.3-13; 2014. Available: http://CRAN.R-project.org/package=boot.

2. Davison AC, Hinkley DV. Bootstrap methods and their applications. Cambridge: Cambridge University Press; 1997.

3. S original, from StatLib and by Rob Tibshirani. R port by Friedrich Leisch. bootstrap: Functions for the Book "An Introduction to the Bootstrap". R package version 2014.4; 2014. Avaliable: http://CRAN.R-project.org/package=bootstrap.

4. Efron B, Tibshirani R. An introduction to the bootstrap. Boca Raton: Chapman & Hall; 1993.
