# Supplementary material for: Speeding Up Non-Parametric Bootstrap Computations for Statistics Based on Sample Moments in Small/Moderate Sample Size Applications
Source: PLoS One. 2015 Jun 30;10(6):e0131333. doi: 10.1371/journal.pone.0131333 (PMC4488363; doi:10.1371/journal.pone.0131333)
Supplement: S2 Text — This file presents a benchmark study comparing single thread “for loop” and vectorized implementations, against parallel “for loop” and vectorized implementations (based on both the parLapply and mclapply alternatives to the lapply function, provided by the parallel R package). (PDF) [file pone.0131333.s002.pdf]

S2 Text. Comparison of single thread versus parallel implementations

As any other bootstrap technique, our vectorized implementation can potentially benefit from parallelization by splitting and distributing the total number of bootstrap calculations across multiple processors/cores. Using the standard capabilities provided in `parallel` R package, we implemented parallel versions of the "for loop" and "vectorized" bootstrap functions for bootstrapping Pearson's correlation coefficient, and we compared the running times of the single threaded versus parallel implementations in all the real data and simulated data examples presented in the main text.

In this benchmark study we evaluated parallel versions based on both the `parLapply` and `mclapply` alternatives to the `lapply` function, in the `parallel` R package. Since forking is not available for the Windows operating system (and the `mclapply` function defaults back to single thread computation in Windows), we benchmarked the results in two distinct platforms, namely, a Intel Core i7-3610QM (2.3 GHz), 24 Gb RAM, Windows 7 Enterprize (64-bit), and a Intel (R) Core (TM) i7 CPU 950@3.07GHz, 24 Gb RAM, Xubuntu 14-04 (64-bit). For the parallel implementations employing the `parLapply` function, we used PSOCK clusters generated by calling `makeCluster(specs, type = "PSOCK")` function (with the `specs` argument set to 4, since both Windows and Xubuntu machines have quad core processors).

Figures 1 to 5 present the results. In all figures, panel a presents the results for the Windows platform, and we see that the timings for the single thread (full lines) and `mclapply` parallel implementations (dotted lines) are very close since forking is not available in Windows and the `mclapply` function defaults back to single thread computation. In all figures, panel b presents the results for the Xubuntu platform. Overall, we observe the following results:

1. Inspection of Figures 3, 4, and 5 shows that, similarly to the results observed in the single thread computations in the main text, the vectorized parallel implementations tended to out-perform the "for loop" parallel implementations in small/moderate sample sizes, but tended to be slower for larger sample sizes.

2. Comparison of the brown curves on Figures 1, 2, 4, and 5 shows that the "for loop" parallel implementations tended to be faster than the "for loop" single thread computations for all sample sizes tested, with the gains being larger for larger sample sizes and number of bootstrap computations. (Note that on Figure 3 ($B = 10,000$), nonetheless, the `parLapply` implementation is slower than single thread computing, although the `mclapply` parallel implementation is faster.)

3. Comparison of the blue curves on all figures shows that the vectorized parallel implementations tended to be faster than the vectorized single thread computations as the sample size and number of bootstrap replications increased (Figures 1, 4 and 5 show that the gap between the single thread and parallel computations tends to increase, as we increase B). However, for small sample sizes the single thread computations were sometimes slightly faster (compare the full and dashed blue lines in Figure 2, and the full and dashed blue lines at small N values on Figure 4).

4. When $B = 10,000$ (Figure 3), comparison of the `parLapply` and `mclapply` implementations (for both "for loop" and vectorized implementations) showed that the `parLapply` function is slower than the single thread computation, whereas `mclapply` is faster. However, for larger numbers of bootstrap replications (Figures 4 and 5) both parallel implementations tend to be faster than the single thread computation, with the `parLapply` becoming more efficient than the `mclapply` as B increases.

Interestingly, these benchmarking results suggest that parallelization should not always be preferred to single thread computation since, sometimes, the time spent in distributing and gathering results across multiple cores can be greater than the time needed for single thread computing. We point out,

nonetheless, that benchmarking results for parallel implementations are highly dependent on hardware specifications.

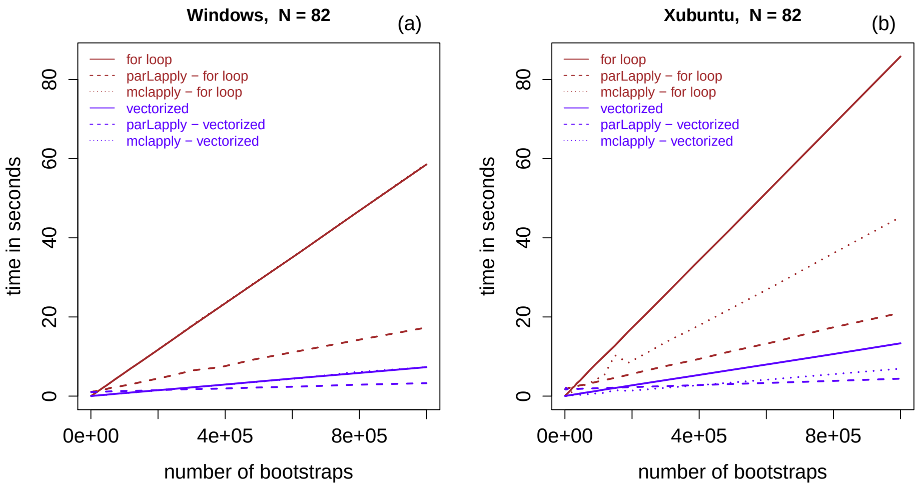

Figure 1. Comparison between the single thread and parallel implementations for the "for loop" (brown) and vectorized (blue) bootstrap approaches, in the American law school data. Panels a and b present the results for the Windows and Xubuntu platforms, respectively. The full line represents the single thread implementation, while dashed and dotted lines represent parallel implementations based on the `parLapply` and `mclapply` functions, respectively.

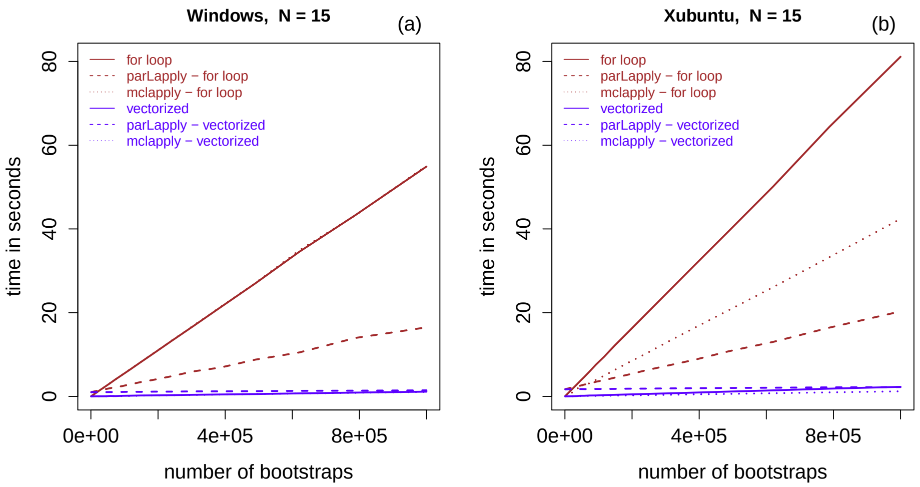

Figure 2. Comparison between the single thread and parallel implementations for the "for loop" (brown) and vectorized (blue) bootstrap approaches, in a subset of the American law school data. Panels a and b present the results for the Windows and Xubuntu platforms, respectively. The full line represents the single thread implementation, while dashed and dotted lines represent parallel implementations based on the `parLapply` and `mclapply` functions, respectively.

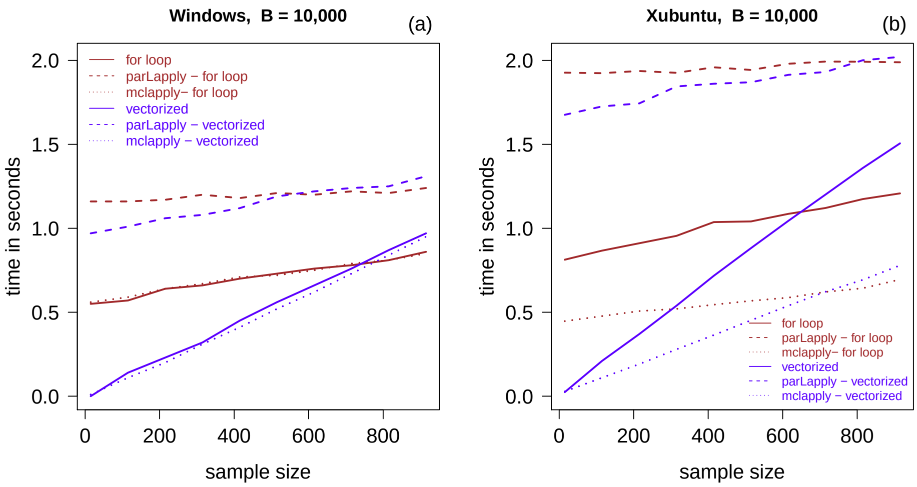

Figure 3. Comparison between the single thread and parallel implementations for the "for loop" (brown) and vectorized (blue) bootstrap approaches, in simulated data using 10,000 bootstrap replications, and sample sizes varying from 15 to 915. Panels a and b present the results for the Windows and Xubuntu platforms, respectively. The full line represents the single thread implementation, while dashed and dotted lines represent parallel implementations based on the `parLapply` and `mclapply` functions, respectively.

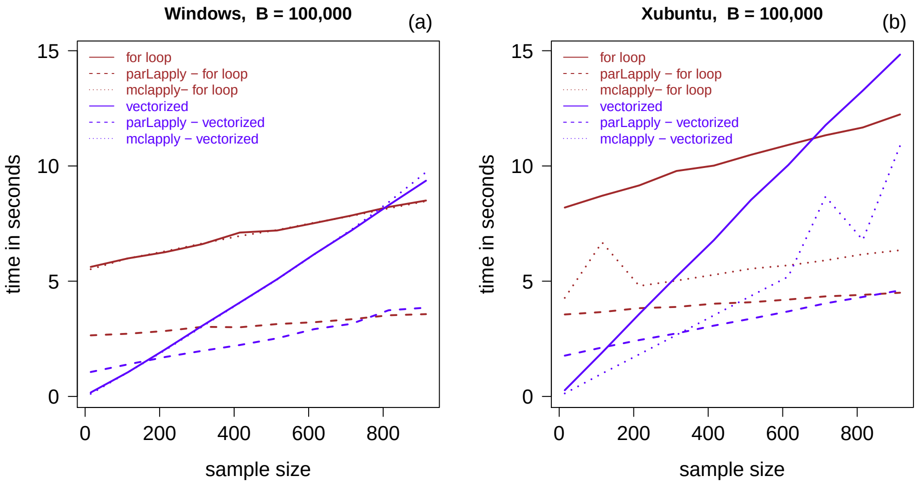

Figure 4. Comparison between the single thread and parallel implementations for the "for loop" (brown) and vectorized (blue) bootstrap approaches, in simulated data using 100,000 bootstrap replications, and sample sizes varying from 15 to 915. Panels a and b present the results for the Windows and Xubuntu platforms, respectively. The full line represents the single thread implementation, while dashed and dotted lines represent parallel implementations based on the `parLapply` and `mclapply` functions, respectively.

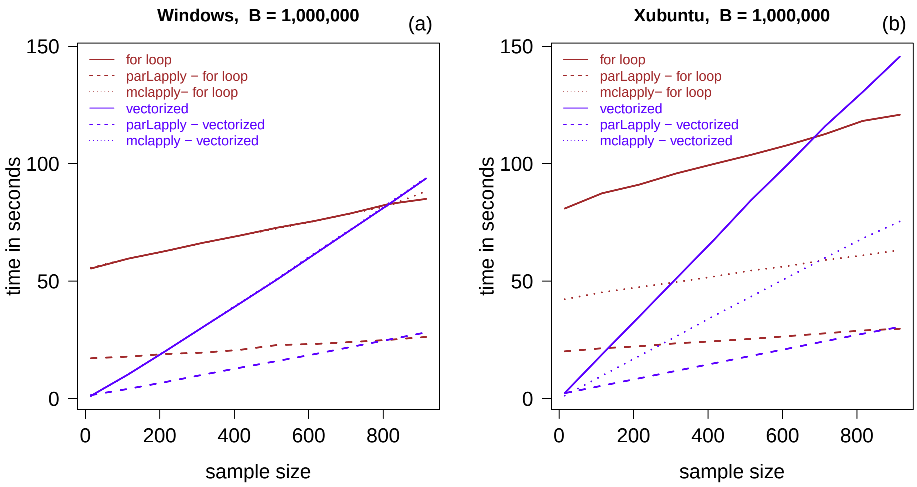

Figure 5. Comparison between the single thread and parallel implementations for the "for loop" (brown) and vectorized (blue) bootstrap approaches, in simulated data using 1,000,000 bootstrap replications, and sample sizes varying from 15 to 915. Panels a and b present the results for the Windows and Xubuntu platforms, respectively. The full line represents the single thread implementation, while dashed and dotted lines represent parallel implementations based on the `parLapply` and `mclapply` functions, respectively.

All the R code used in the generation of these results is available at `https://raw.githubusercontent.com/echaibub/VectorizedNonParametricBootstrap/master/run_cor_parallel_comparisons.R`
