# Supplementary material for: Speeding Up Non-Parametric Bootstrap Computations for Statistics Based on Sample Moments in Small/Moderate Sample Size Applications
Source: PLoS One. 2015 Jun 30;10(6):e0131333. doi: 10.1371/journal.pone.0131333 (PMC4488363; doi:10.1371/journal.pone.0131333)
Supplement: S3 Text — This file presents a benchmarking study comparing the vectorized bootstrap based on multinomial frequencies against a vectorized implementation of the approximate bootstrap based on Poisson frequencies. (PDF) [file pone.0131333.s003.pdf]

S3 Text. Comparison of the multinomial and Poisson frequencies bootstraps.

Hanley and MacGibbon [1] proposed the use of Poisson frequencies to approximate the non-parametric bootstrap. The basic idea is that, instead of sampling bootstrap frequencies $\boldsymbol{n}^* = (n_1^*, \ldots, n_N^*)^t$ from a Multinomial$(N, N^{-1}\mathbf{1}_N)$ distribution, one can generate N random frequencies, $(p_1^*, \ldots, p_N^*)$, from a Poisson distribution with expectation 1. Whereas in the multinomial version the sum of the bootstrap frequencies, $\sum_{i=1}^N n_i^*$, is always N, the Poisson frequencies do not sum to N, although the expectation of $\sum_{i=1}^N p_i^*$ is N. In other words, the multinomial bootstrap generates samples of fixed size N, while the Poisson approach generates samples with variable sizes (but equal to N, on average). For instance, when bootstrapping the mean, $\bar{y} = N^{-1} \sum_{i=1}^N y_i$, the multinomial approach uses $\bar{y}^* = N^{-1} \sum_{i=1}^N n_i^* y_i$, whereas the Poisson frequencies approach uses $\bar{y}^* = (\sum_{i=1}^N p_i^* y_i)/(\sum_{i=1}^N p_i^*)$. The authors show that the approximation improves as N increases, but is already reasonable for moderate sample sizes. Clearly, this approximate approach can also be easily vectorized.

In order to check whether sampling from a Poisson distribution could lead to a computationally more efficient vectorized bootstrap (and also evaluate the quality of the approximation) we benchmarked the Poisson frequencies approach against the multinomial sampling bootstrap. Following the real data example in the main text, we bootstrapped Pearson's sample correlation coefficient over a grid of bootstrap replications, ranging from 10,000 to 1,000,000, using the full set ($N = 82$), as well as, a subset ($N = 15$) of the American law schools data (pages 17 and 19 of [2]). The results are presented on Figure 1.

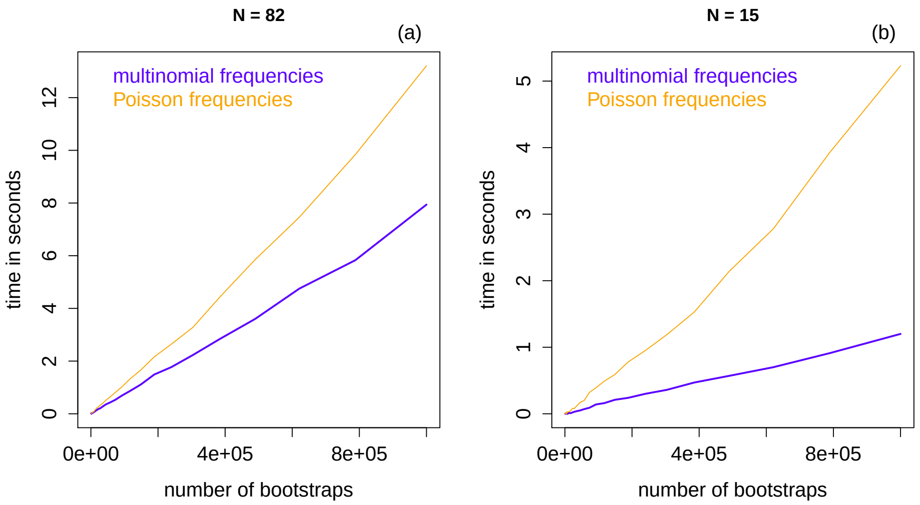

Figure 1. Comparison of vectorized implementations based on Poisson (orange curves) and multinomial (blue curves) frequencies, in the American law school data (panel a) and in a subset of the American law school data (panel b).

Figure 1 shows that the multinomial frequencies approach is slightly faster than the Poisson alternative. The likely reason for this difference is that in the generation of the weight matrix with the multinomial sampling approach, we only need to divide each multinomial frequency vector by the sample size N (line 3 in the code snippet pasted below), while in the generation of the Poisson frequencies we first need to compute the sum of each Poisson frequency vector (line 9), and then divide each frequency

vector by its sum (line 10). These extra computation steps seem to explain the longer time taken by the Poisson frequencies approach.

```
1  BootWeights <- function(N, B) {
2    counts <- rmultinom(B, N, rep(1/N, N))
3    counts/N
4  }
5
6  BootPoissonWeights <- function(N, B) {
7    counts <- rpois(N * B, lambda = 1)
8    counts <- matrix(counts, B, N)
9    aux <- apply(counts, 1, sum)
10   counts/aux
11 }
```

Figure 2 compares the approximate bootstrap distributions generated with Poisson frequencies against the bootstrap distributions generated via multinomial sampling (using again the full set, $N = 82$, and the subset, $N = 15$, of the American law schools data). The top panels show that the Poisson frequencies approximation works well when $N = 82$. The bottom panels, on the other hand, suggest that this is not the case for $N = 15$ (note the discrepancy of the distributions tails, clearly shown by the quantile-quantile plot in panel f).

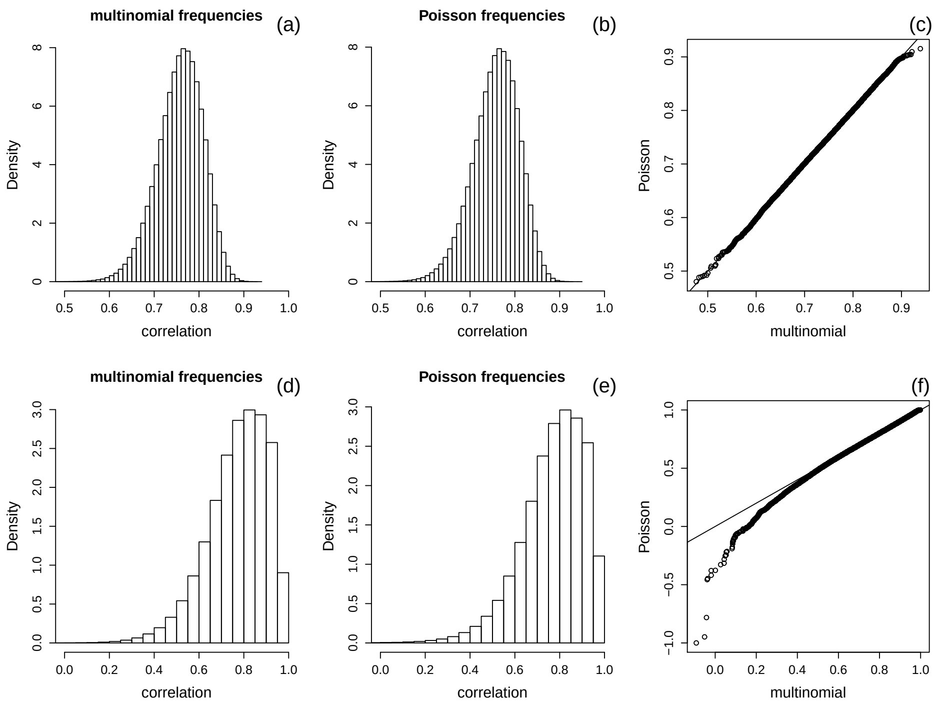

Figure 2. Comparison of the multinomial sampling non-parametric bootstrap versus the approximate bootstrap based on Poisson frequencies. Top panels show the results for the American law school data ($N = 82$). Bottom panels used a subset of the American law school data ($N = 15$). Results based on $B = 1,000,000$.

All the timings in this study were measured on an Intel Core i7-3610QM (2.3 GHz), 24 Gb RAM,

Windows 7 Enterprize (64-bit) platform. The R code used in the generation of these results is available at

`https://raw.githubusercontent.com/echaibub/VectorizedNonParametricBootstrap/master/run\_multinomial\_vs\_Poisson\_boot\_comparison.R}.`

References

1. Hanley JA, MacGibbon B. Creating non-parametric bootstrap samples using Poisson frequencies. Computer Methods and Programs in Biomedicine. 2006; 83: 57-62.

2. Efron B, Tibshirani R. An introduction to the bootstrap. Boca Raton: Chapman & Hall; 1993.
